# Non-Surgical Strategies for Managing Skeletal Deformities in a Child with X-Linked Hereditary Hypophosphatemic Ricket: Insights and Perspectives

**DOI:** 10.3390/children11040487

**Published:** 2024-04-18

**Authors:** Tung-Hee Tie, Wei-Han Lin, Ming-Tung Huang, Po-Ting Wu, Meng-Che Tsai, Yen-Yin Chou, Chih-Kai Hong, Chii-Jeng Lin, Chien-An Shih

**Affiliations:** 1 Department of Orthopedics, National Cheng Kung University Hospital, College of Medicine, National Cheng Kung University, Tainan 704, Taiwan; n106077@mail.hosp.ncku.edu.tw (T.-H.T.); ttoonnyyhuang@gmail.com (M.-T.H.); z10508032@ncku.edu.tw (P.-T.W.); yayahong@gmail.com (C.-K.H.); mark@mail.ncku.edu.tw (C.-J.L.); 2 Medical Device R & D Core Laboratory, National Cheng Kung University Hospital, Tainan 704, Taiwan; 3 School of Medicine, National Cheng Kung University, Tainan 701, Taiwan; i54076199@gs.ncku.edu.tw; 4 Department of Orthopedics, College of Medicine, National Cheng Kung University, Tainan 701, Taiwan; 5 Department of Biomedical Engineering, National Cheng Kung University, Tainan 701, Taiwan; 6 Department of Biochemistry and Molecular Biology, College of Medicine, National Cheng Kung University, Tainan 701, Taiwan; 7 Department of Pediatrics, National Cheng Kung University Hospital, College of Medicine, National Cheng Kung University, Tainan 704, Taiwan; n041427@mail1.hosp.ncku.edu.tw (M.-C.T.); yenyin@mail.ncku.edu.tw (Y.-Y.C.); 8 Department of Orthopedic Surgery, Show Chwan Memorial Hospital, Changhua 500, Taiwan

**Keywords:** X-linked hypophosphatemic rickets, non-surgical treatment, skeletal deformity, therapeutic orthotics, bone alignment

## Abstract

This case report sheds light on the management of skeletal deformity in a young child with X-linked hypophosphatemia (XLH), emphasizing the significance of a timely orthotic intervention alongside pharmacological treatment, which is a strategy not frequently highlighted in the XLH literature. The patient, a 2-year-and-7-month-old female, presented with classic XLH symptoms, including short stature, pronounced genu varum, and hypophosphatemia, with deformities observed in both the coronal and sagittal planes of the femur and tibia. Despite initial reliance on pharmacotherapy, which proved insufficient for skeletal realignment, the integration of orthotic treatment at age 3 marked a pivotal turn in the management strategy. By the age of 5 years and 9 months, this combined approach yielded significant improvements: the deformities in the femur and tibia were notably corrected, tibial torsion was addressed, and enhanced limb alignment was achieved, as corroborated by radiographic evidence. This case underscores the effectiveness of orthotic intervention as a critical and underemphasized adjunct to pharmacological therapy in managing XLH in early childhood. It advocates for the early inclusion of orthotic measures to optimize treatment outcomes and expand the range of management strategies for limb deformities.

## 1. Introduction

X-linked hypophosphatemia (XLH) is a rare metabolic disorder associated with progressive rickets (XLHR), severe deformities, and osteomalacia, resulting from a loss-of-function mutation in the PHEX gene on the X chromosome [1,2]. This mutation leads to the dysregulation of fibroblast growth factor 23 (FGF23), causing chronic renal phosphate wasting and influencing the activation of 1,25-dihydroxyvitamin D [1,25(OH)2D] [1,3].

Patients with XLH commonly exhibit rachitic deformities in the lower limbs, which contribute to short stature due to sustained phosphate deficiency and its impact on growth plate development [2,4,5]. Common deformities include bilateral genu varum with internal tibial torsion and bilateral genu valgum [6,7]. Pediatric hypophosphatemic rickets management aims to control hypophosphatemia, prevent long bone deformities, promote normal growth and minimize bony lesions [3]. Initial treatments often include vitamin D or its analogs and oral phosphate supplementation, though their efficacy varies [2,3,8]. The effectiveness of knee–ankle–foot orthoses in XLH treatment remains a topic of debate [4,8]. When varus deformity persists or worsens despite medical treatment, surgical interventions are considered, employing guided growth for mild cases and osteotomies with internal fixation for more severe cases unresponsive to guided growth [6]. However, osteotomies in early childhood carry a high risk of recurrence and complications [4].

This case report presents our approach to managing a young patient with XLH who exhibited severe bowleg deformity. Initially managed with conventional therapy, the patient’s transition to orthotic treatment was pivotal when conventional methods alone failed to correct mechanical alignment. This report highlights the successful correction achieved through orthotic management with diligent compliance, thereby avoiding surgery. It underscores the importance of timely intervention and the role of orthosis, documenting detailed changes in skeletal coronal, sagittal, and torsional alignment, as well as radiographic improvements, to demonstrate the effects of the combined orthotic treatment.

## 2. Case Description

### 2.1. Diagnosis

**Figure 1:**
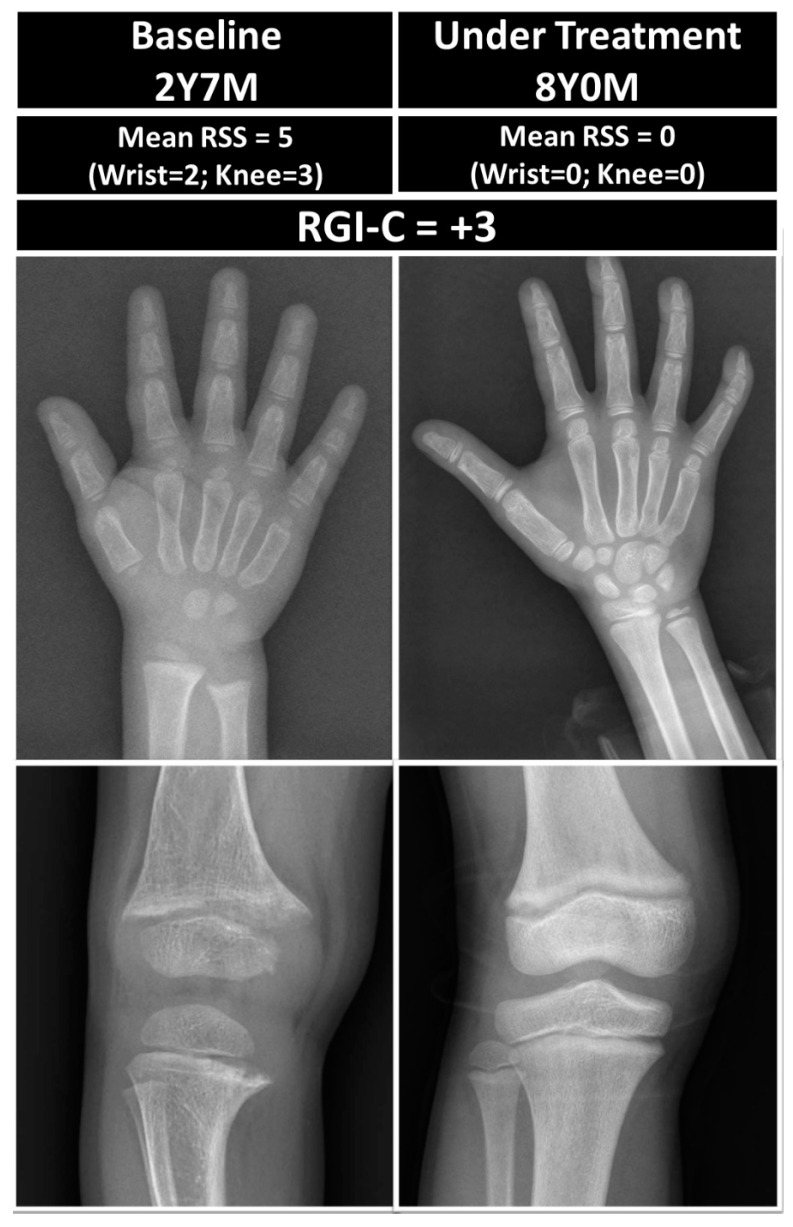
Comparative radiographs and assessment from baseline to 5 years and 6 months post treatment using mean Rickets Severity Score (RSS) and Radiographic Global Impression of Change (RGI-C).

A girl aged 2 years and 4 months, with a height of 76.7 cm (<3rd percentile) and a weight of 9.1 kg (<3rd percentile), was presented to our pediatric endocrinology and orthopedic outpatient clinic due to concerns regarding short stature, progressive bowleg, and bilateral knee pain. There was no notable family history of X-linked hypophosphatemia (XLH) or short stature in her immediate family. Laboratory evaluations indicated hypophosphatemia, elevated alkaline phosphatase (ALP), diminished 1,25-dihydroxyvitamin D (1,25(OH)2D) levels, a decreased renal tubular threshold for phosphate (TmP/GFR), and reduced urine calcium excretion (urine Ca/Creatinine ratio) (Table 1). Radiographic assessments revealed abnormalities in the distal femur, proximal tibia, and distal radius and ulna, characterized by cupping, fraying, and physeal widening [9] (Figure 1). Genetic analysis identified a mutation in the PHEX gene (exon 8, C.931C > T), confirming a diagnosis of X-linked hypophosphatemia rickets. This case study received approval from the National Cheng Kung University Hospital Institutional Review Board (IRB No. B-EC-113-012).

### 2.2. Treatment Transition

**Figure 2:**
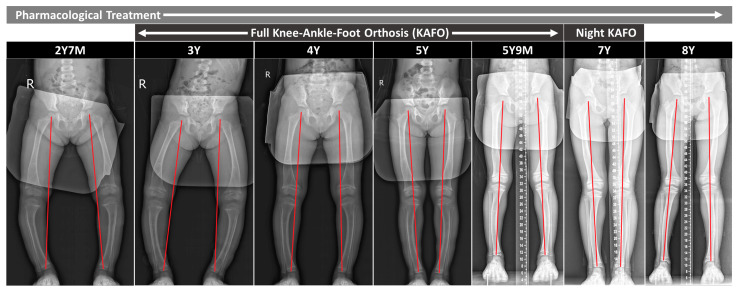
Scanograms illustrating mechanical axis deviation during initial pharmacological therapy (age 2 years 7 months), post combination orthotic management (age 3 years to 7 years), and at final evaluation (age 8 years).

The patient commenced treatment at the Pediatric Endocrine and Orthopedics Surgery clinic at the age of 2 years and 7 months. The regimen included oral phosphate (20–60 mg/kg/day [3], starting dose of 25 mg/kg/day) and active vitamin D—Calcitriol (20–30 ng/kg/day [6], starting dose of 25 ng/kg/day), with adjustments based on serum phosphorus levels until the age of 8 (Table 1). Despite six months of pharmacological intervention, her bowleg deformity progressed, necessitating the implementation of a full-time knee–ankle–foot orthosis (KAFO) [4] at 3 years of age (Figure 2). This orthosis was maintained until she achieved appropriate mechanical alignment at 5 years and 9 months, followed by nighttime bracing until age 7, which was then discontinued due to substantial improvement in lower limb alignment (Figure 2).

### 2.3. Skeletal Profile Changes

#### 2.3.1. Coronal Alignment

During the initial phase of pharmacological treatment, a majority of the coronal alignment parameters showed deterioration; however, upon initiating orthosis therapy, these parameters exhibited improvement (Figure 2). Complete correction was observed in the mechanical axis and mechanical axis deviation (MAD) [6], accompanied by notable improvements in the angles of the femur and tibia (Table 2).

#### 2.3.2. Femur/Tibia Bow and Metaphyseal-Diaphyseal Angle

**Figure 3:**
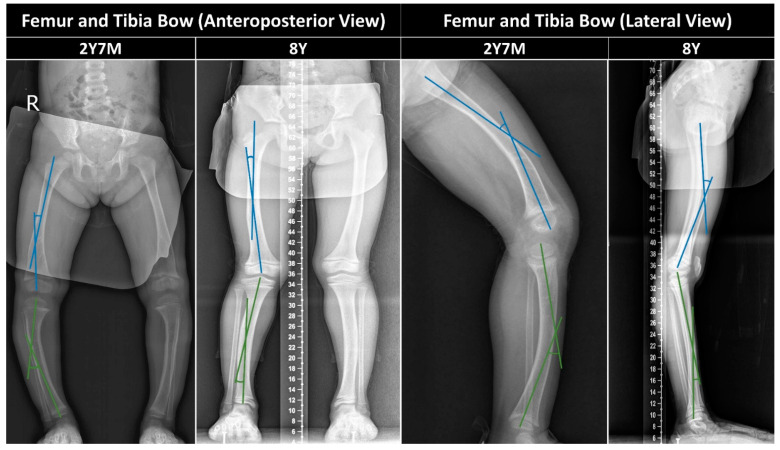
Anteroposterior and lateral views highlighting femoral (blue lines) and tibial (green lines) bows.

**Figure 4:**
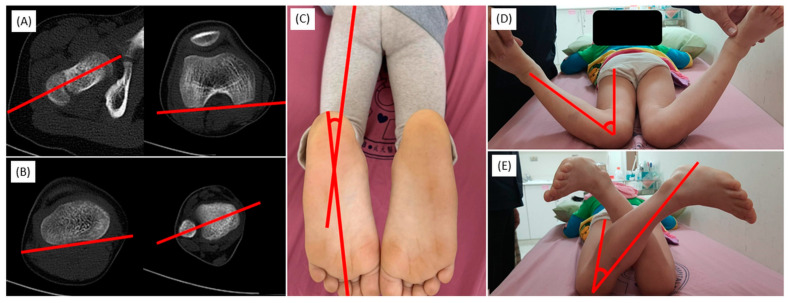
CT scans demonstrating femoral anteversion (**A**), tibial internal torsion (**B**), with clinical evaluation of thigh–foot angle (**C**), hip external rotation (**D**) and internal rotation (**E**).

Improvements in the coronal tibia bow and the metaphyseal-diaphyseal angle (MDA) [5] were noted with the start of pharmacological treatment, and these improvements were enhanced with the introduction of orthosis therapy (Figure 2 and Figure 3 and Table 2). Although the coronal bowing of the femur initially worsened with pharmacological treatment alone, it showed enhancement with the addition of orthosis therapy (Table 2). Additionally, significant improvements were observed in the sagittal bowing of the femur and tibia (Table 2 and Figure 4).

#### 2.3.3. Axial Alignment

At 2 years and 7 months of age, the patient demonstrated an in-toeing gait characterized by an average negative thigh–foot angle (TFA) [6] of 28°. This angle showed considerable improvement by the age of 7, with an average TFA of 10°. Computed tomography scans revealed femoral anteversion with angles of 21° (right) and 20° (left), and tibial internal torsion with angles of 13° (right) and 7° (left), confirming significant corrections in axial alignment (Figure 4).

#### 2.3.4. Radiographic Grades and Healing

The Rickets Severity Score (RSS) [4] saw an improvement from five to zero by the age of 8. Additionally, the Radiographic Global Impression of Change (RGI-C) score [10] was recorded at +3 (Figure 1).

#### 2.3.5. Growth Chart and Bone Age

**Figure 5:**
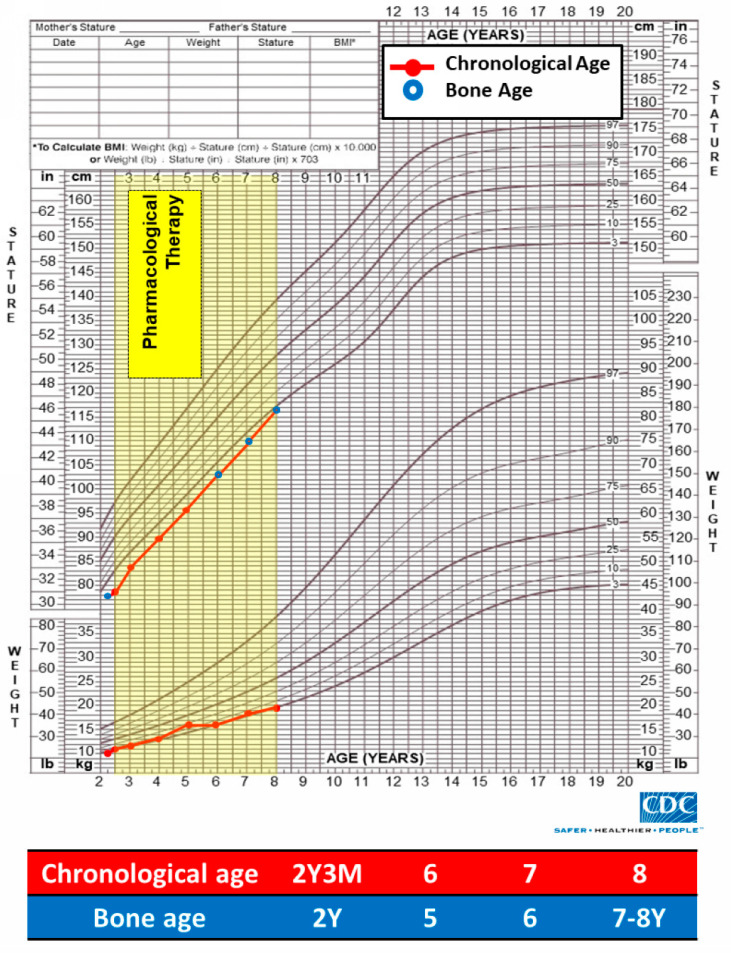
Growth curve displaying changes in height, weight and bone age before and after pharmacological intervention.

Despite the patient’s height and weight remaining below the third percentile post treatment, there was an observable trend toward catch-up growth, with height approaching the third percentile by age 8. The bone age was consistent with the chronological age of the patient (Figure 5).

## 3. Discussion

Lower limb deformities, such as gait alterations and pain, are prevalent complaints among patients with X-linked hypophosphatemic rickets (XLHR), affecting both children and adults and impacting their quality of life. In this report, we discuss a 2-year-7-month-old female with XLHR who presented with genu varum, as well as bowing of the femur and tibia in both coronal and sagittal planes, accompanied by significant in-toeing. Initially, she was treated with conventional pharmacologic therapy, which was followed by the integration of orthotic treatment at the age of 3 years due to an inadequate response to the initial treatment. This combined approach successfully corrected the mechanical alignment and improved the deformities in the femur and tibia, as well as tibia torsion, alongside radiographic healing. This case is notable as it provides detailed insights into the efficacy of orthotic treatment in managing XLHR in early childhood.

The primary objective of non-surgical management for X-linked hypophosphatemic rickets (XLHR) limb deformities is to enhance growth, development and gait while correcting deformities [2]. Traditional approaches have relied heavily on oral phosphate supplements and active vitamin D [4,5]. The overarching goal is the resolution of rickets in children, coupled with efforts to diminish skeletal deformities, promote growth, alleviate bone pain, and potentially avoid surgical interventions [5]. Studies have demonstrated that conventional treatment elevates serum phosphate levels and decreases both osteoid thickness and volume, which significantly correlates with reduced symptoms of bone and joint pain [2]. Effective phosphate management through these treatments may reduce the necessity for orthopedic surgeries [2,4]. Nonetheless, variability in treatment response is noted, particularly in severe XLHR cases where radiographic improvement in rickets does not always correspond with corrections in leg bowing or deformities [2,7].

Burosumab, an anti-fibroblast growth factor 23 antibody, has gained regulatory approval for the treatment of XLHR [5,11]. Initially approved by the European Medicines Agency and the U.S. Food and Drug Administration in 2018 for use in children over one year of age—and subsequently for infants as young as six months—the treatment has also been authorized for adults in the EU as of 2020 [2]. Current guidelines suggest initiating Burosumab for patients with radiographically confirmed rickets that is resistant to standard treatments or in those who cannot tolerate or experience complications from conventional therapies [2]. While Burosumab has shown promising outcomes in reducing bone deformities and enhancing growth, comprehensive studies comparing its efficacy specifically for bone deformities are currently limited, focusing mostly on lower limb deformity scores [5,12]. However, some evidence indicates that Burosumab may not significantly impact lower limb deformities after one year of treatment [4]. Future research is essential to evaluate both the short- and long-term comparative effects of Burosumab against traditional treatments for correcting bone deformities.

Orthotic management, particularly the use of knee–ankle–foot orthoses, may represent an adjuvant non-operative strategy for addressing lower limb deformities in XLHR in early childhood [4,13]. However, evidence of the efficacy of such orthoses is sparse and derives mostly from studies on vitamin-D-resistant rickets, yielding mixed results [4]. A study from Japan noted that orthotic treatment was effective in a limited cohort of rachitic patients, including those with XLHR [13]. In cases where varus deformities persist or worsen despite medical management, orthopedic specialists may consider orthotic management or opt for surgical interventions like guided growth or corrective osteotomies [2,6]. There remains no consensus on prioritizing orthotic over surgical treatments, and the effectiveness of orthosis in managing rickets continues to be debated, with studies reporting conflicting outcomes [4,8,13]. Our findings demonstrate that while the initial pharmacological treatment only addressed the coronal tibia bow and medial distal angle (MDA), the introduction of orthotic treatment yielded significant improvements in both coronal and sagittal alignments, as well as rotational adjustments, aligning with previous studies that suggest conventional therapy can improve coronal alignment but may not effectively correct rotational deformities [2,4,6].

In late childhood and adolescence, when deformities such as severe varus, valgus, or windswept lower leg deformities become more pronounced, treatment often involves surgical interventions [4,14]. Surgical interventions like guided growth surgery offer a less invasive option for correcting coronal plane deformities, allowing patients a quicker return to normal activities [3,4,6,7,8,10]. However, mostly in cases of severe deformities, such procedures may not provide sufficient correction, necessitating more intensive surgeries involving osteotomies with internal or external fixation [4,6]. Earlier surgical interventions, while addressing the deformities, are associated with high rates of recurrence and complications, and some studies have reported a recurrence rate as high as 90% after the initial corrective osteotomy, despite adequate phosphate management [6]. Moreover, these surgical procedures carry the risk of iatrogenic physeal disruption [4].

This study has limitations, including its focus on a single case, highlighting the need for further larger-scale research to assess the effectiveness of orthotic treatment broadly. Additionally, while Burosumab [5,11] emerges as a promising treatment alternative, its unavailability in our region calls for future research to evaluate its efficacy in conjunction with conventional or combined orthotic therapies.

## 4. Conclusions

This case underscores the effectiveness of orthotic intervention as a critical and underemphasized adjunct to pharmacological therapy in XLH, advocating for its early inclusion to optimize treatment outcomes and broaden the spectrum of management strategies for limb deformities.

## Figures and Tables

**Table 1:** Biochemical profile at diagnosis and throughout pharmacological treatment.

	Pharmacological Treatment (2 y 7 m–8 y)							Reference Range
	2 y 7 m (Diagnosis Age)	3 y	4 y	5 y	6 y	7 y	8 y (Current Age)	
ALP(U/L)	635	372	456	416	362	415	388	1–10 yrs old: 156–369
Ca(mmol/dL)	2.31	2.25	2.37	2.27	2.27	2.34	2.29	1–5 yrs old: 2.35–2.70 6–12 yrs old: 2.35–2.57
P(mmol/dL)	0.97	0.91	0.97	1.59	1.10	1.17	0.97	1–5 yrs old: 1.05–1.95 6–12 yrs old: 1.00–1.80
25(OH)D (ng/mL)	16.1	NA	NA	NA	26.4	NA	NA	1–3 yrs old: 45–145 3–19 yrs old: 43–98
Intact PTH (pg/mL)	60	39.5	26.2	50.8	40.9	27.1	35.7	10–65
U-Ca/U-Cre (mg/mg)	0.06	NA	NA	NA	0.22	0.19	0.13	1–3 yrs old: 0.03–0.56 3–5 yrs old: 0.02–0.41 5–7 yrs old: 0.01–0.30 7–10 yrs old: 0.01–0.25
TmP/GFR mmol/dL)	0.78	NA	NA	NA	NA	NA	NA	1–5 yrs old: 1.05–1.78 6–12 yrs old: 0.97–1.64
TRP	0.84	NA	NA	NA	NA	NA	NA	0.85–0.95
IGF-1 (ng/mL)	111	NA	NA	NA	NA	NA	NA	51–218
Phosphorus (mg/kg/day)	25	23	55	44	57	49	55	20–60 mg/kg/day
Dihydroxycholecalciferol (mg/kg/day)	25	24	29	31	31	27	26	20–30 ng/kg/day

NA, not available; ALP, alkaline phosphatase; Ca, calcium; P, phosphate; 25(OH)D, 25-hydroxyvitamin D; PTH, parathyroid hormone; U-Ca/U-Cre, ratio of urine calcium to creatine; U-P/U-Cre, ratio of urine phosphate to creatine; TmP/GFR, ratio of tubular maximum reabsorption rate of phosphate to glomerular filtration rate; TRP, tubular reabsorption of phosphorus.

**Table 2:** Sequential radiographic changes across different treatment phases.

		Conventional Therapy (2 y 7 m–8 y)								
			Orthosis Therapy (3 y–7 y)						Mean Correction Rate †	
		2 y 7 m	3 y	4 y	5 y	5 y 9 m	7 y	8 y	Before Orthosis (2 y 7 m–3 y)	During Orthosis (3 y–7 y)
Mechanical axis (°) *	R	24	26	10	3	0	−2	−3	+4°/year	−7°/year
	L	20	27	11	3	0	−1	−2		
Mechanical axis deviation (mm) *	R	35	40	17	4	0	−4	−0.5	+15 mm/year	−10.5 mm/year
	L	28	38	16	5	0	−2	−0.6		
mLPFA (°)	R	74	73	69	73	69	81	83	−8.0°/year	+2.6°/year
	L	72	65	65	68	68	78	82		
mLDFA (°)	R	96	97	94	91	89	89	88	+1.0°/year	−1.9°/year
	L	96	96	97	93	90	89	87		
mMPTA (°)	R	70	73	80	87	90	90	91	+5.0°/year	+3.8°/year
	L	75	77	81	87	90	90	90		
mLDTA (°)	R	94	93	92	86	88	90	90	+1.0°/year	−1.0°/year
	L	93	95	90	86	88	90	90		
Metaphyseal-diaphyseal angle (°)	R	19	13	7	2	1	−2	−1	−9°/year	−3.5°/year
	L	14	11	7	2	1	−2	−1		
Coronal femoral diaphyseal bow (°)	R	16	20	16	16	15	14	7	+10°/year	−2.3°/year
	L	14	20	16	13	10	8	8		
Coronal tibia diaphyseal bow (°)	R	28	23	23	19	17	10	8	−5.0°/year	−4.0°/year
	L	29	29	26	21	14	10	8		
Sagittal femoral diaphyseal bow (°)	R	31	NA	NA	NA	NA	18	6	−3.5°/year ‡	
	L	31	NA	NA	NA	NA	20	18		
Sagittal tibia diaphyseal bow (°)	R	25	NA	NA	NA	NA	8	2	−4.1°/year ‡	
	L	28	NA	NA	NA	NA	6	6		

NA, not available; R, right; L, left; y, year; m, month; mLPFA: mechanical lateral proximal femoral angle; mLDFA, mechanical lateral distal femoral angle; mMPTA, mechanical medial proximal tibial angle; mLDTA, mechanical lateral distal tibial angle. * +: varus; −: valgus. † +: positive correction; −: negative correction. ‡: during whole treatment period.

## Data Availability

The data presented in this study are available in the article.
